# Enhanced photocatalytic CO_2_ conversion of a CdS/Co-BDC nanocomposite *via* Co(ii)/Co(iii) redox cycling†

**DOI:** 10.1039/d4ra04842c

**Published:** 2024-08-13

**Authors:** Ruina Xuan, Jieqiong Mo, Jiwen Chen, Yixin Dou, Xiaofang Li, Zhuo jiang, Bo Chai, Chunlei Wang, Deng Ding, Juntao Yan, Xiaobo Wang

**Affiliations:** a College of Chemistry and Environmental Engineering, Wuhan Polytechnic University Wuhan 430023 China dingdeng@whu.edu.cn +86-15927216471; b School of Electrical Engineering and Automation, Wuhan University Wuhan 430072 China

## Abstract

Photocatalytic CO_2_ reduction into value-added chemical fuels using sunlight as the energy input has been a thorny, challenging and long-term project in the environment/energy fields because of to its low efficiency. Herein, a series of CdS/Co-BDC composite photocatalysts were constructed by incorporating CdS nanoparticles into Co-BDC using a dual-solvent *in situ* growth strategy for improving photocatalytic CO_2_ reduction efficiency. The composites were characterized through XRD, SEM, TEM, XPS, DRS and EPR techniques in detail. 18% CdS/Co-BDC composites showed superior performance for the photocatalytic CO_2_ reduction to CO, which was 8.9 and 19.6 times higher than that showed by the pure CdS and Co-BDC, respectively. The mechanism of enhanced photocatalytic CO_2_ reduction performance was analyzed. The CdS/Co-BDC composites showed better adsorption for CO_2_. Detailed analysis of XPS, transient photocurrent responses, and electrochemical impedance spectroscopy (EIS) shows the existence of strong charge interaction between CdS and Co-BDC and the photo-electrons of CdS can be transferred to Co-BDC. Additionally, Co-oxo of Co-BDC plays the role of a redox-active site and promotes the reduction performance *via* the method of valence transition of Co(ii)/Co(iii) redox.

## Abstract 2

Photocatalytic CO_2_ reduction into value-added chemical fuels using sunlight as the energy input has been a thorny, challenging and long-term project in the environment/energy fields because of to its low efficiency.

## Graphical abstract

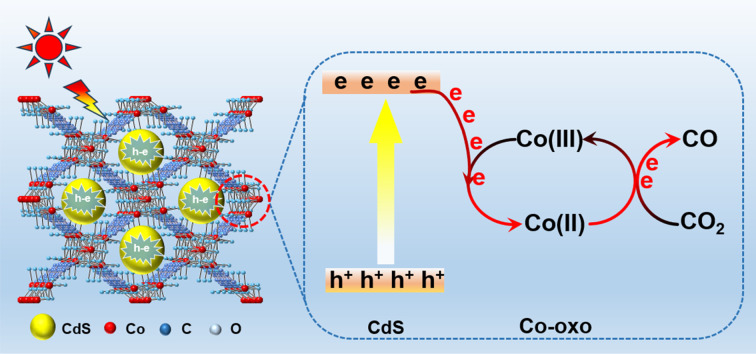

## 1. Introduction

The growing consumption of mineral fuel has expedited the exhaustion of limited natural resources and caused excessive CO_2_ emission into the atmosphere.^1^ Therefore, developing practical pathways to reduce CO_2_ emissions is essential. In the past decades, researchers have investigated various physical, chemical, and biological methods to relieve the energy crisis and the increased panic from climate change and sea-level rising. Owing to the stable C=O bond of the CO_2_ molecule and its dissociation energy (750 kJ mol^−1^),^2^ direct chemical conversion of CO_2_ necessitates the provision of a significant activation energy from the external environment in addition to the removal of an enormous thermodynamic energy barrier; thus, a substantial energy intake is necessary. Photocatalysis can efficiently utilize solar energy to convert it into hydrocarbon fuel *via* a “holy grail” reaction. Based on this, a photocatalytic CO_2_ reduction system is considered an ideal measure to tackle energy depletion and environmental issues. Photocatalytic CO_2_ conversion to CO is currently attracting a lot of attention as an environmentally benign strategy for curbing anthropogenic CO_2_ emission while delivering commodity chemicals.

Since the first report on the photocatalytic reduction of CO_2_ into organic compounds on TiO_2_,^3^ multitudinous photocatalysts with suitable energy band structures have been developed, including TiO_2_, CdS,^4^ g-C_3_N_4_,^5^ Bi_2_WO_6_ (ref. 6) and metal–organic frameworks (MOFs). Among these photocatalysts, the n-type semiconductor CdS with a narrow band gap (*E*_g_ = 2.43 eV) is a well-researched photocatalyst for directly converting solar energy into chemical fuels through water splitting or organic photosynthesis.^7,8^ Nevertheless, CdS particles as a photocatalyst have some drawbacks that result in a low efficiency of CO_2_ reduction: the primary challenges are photo-corrosion of CdS particles, high recombination rate of photo-generated carriers and unregulated product selectivity. Nowadays, the construction of high-performance CdS-based photocatalysts remains an urgent problem to resolve, despite attempts to modify the morphology of CdS and its composites with semiconductors, noble metals, porous materials, and others.^9^ Optimally, heterogeneous structures should not only enhance the photostability of CdS but also provide sites with a high density that can efficiently receive photo-excited electrons.

Metal–organic skeletons (MOFs) are a class of porous crystalline materials composed of metal ions and organic ligands through coordination self-assembly. In recent years, due to their unique structures and properties, including high porosity, low density, large specific surface area, tunable pore size, and topological diversity, MOFs have shown great potential in the field of energy storage and conversion, making them one of the hotspots in today's research. For example, NH_2_-MIL-53(Fe), NH_2_-MIL-88B(Fe), NH_2_-MIL-101(Fe), and NH_2_-UiO-66 have been widely used for photocatalytic conversion of CO_2_.^10^ Compared with conventional inorganic photocatalysts, a large number of catalytically active sites exist in MOF materials. Yan *et al.* combined TiO_2_ with Co-ZIF-9, which enhanced the separation efficiency of photogenerated electrons and holes and improved the performance of photocatalytic CO_2_ reduction.^11^ Qin *et al.* successfully synthesized a dodecahedral rhombic micropore crystal ZIF-67 by using a simple co-precipitation method at room temperature, and the CO generation rate of the photocatalytic CO_2_ reduction system was 37.4 μmol/30 min under the optimal reaction conditions.^12^ Dong *et al.* successfully prepared a series of novel Co-MOF/Cu_2_O (*x*CMC) composites by employing a stepwise self-assembling strategy, and the synthesized 33 wt% of the CMC hybrid material exhibited the maximum photocatalytic activity, which can photocatalytically reduce CO_2_ to CO with a selectivity close to 100%.^13^ Therefore, the construction of Co-based MOF composites to improve photocatalytic performance is very promising.

Herein, Co-MOF (Co-BDC) was synthesized by a hydrothermal method, and a series of different contents of CdS/Co-BDC composites were obtained by a double-solvent method of *in situ* growth of CdS into Co-BDC caves. The characteristics of the CdS/Co-BDC composites, such as the chemical composition and morphological structure, were analyzed using various characterization means. The photocatalytic CO_2_ reduction performance of the CdS/Co-BDC composites was evaluated using CO as the main product and the stability of the composites was probed. The mechanisms of the photocatalytic CO_2_ reduction in the CdS/Co-BDC composites were analyzed by XPS, EPR and electrochemical tests.

## 2. Experimental

### 2.1. Materials

Cobalt(ii) nitrate hexahydrate (Co(NO_3_)_2_·6H_2_O, 99.99%) and cadmium nitrate tetrahydrate (Cd(NO_3_)_2_·4H_2_O, 99%) were purchased from Aladdin Biochemical Science and Technology Co. Ltd, terephthalic acid (1,4-BDC, 99%) was purchased from Shanghai McLean Biochemistry and Technology Co. Ltd, thioacetamide (TAA, 99%), *N*,*N*′-dimethylformamide (DMF, 99.5%), *n*-hexane (C_6_H_14_, 97%), and anhydrous ethanol (EtOH, 99.8%) were purchased from Sinopharm Chemical Reagent Co. Ltd, and triethylamine (TEA, 99%) was purchased from Chengdu Kelon Chemical Co. Ltd. All the chemicals were used as-received and did not require further purification.

### 2.2. Sample preparation

Co-BDC was synthesized following previous literature.^14^ Firstly, Co (NO_3_)_2_·6H_2_O (1.23 g, 4.226 mmol) and 1,4-BDC (0.48 g, 2.889 mmol) were dissolved in 200 mL of DMF. After stirring for 1 h, 20 mL of water, 20 mL of ethanol and 10 mL of triethylamine (TEA) were added to the above solution. The solution continued to be stirred at room temperature for 12 h. Finally, the product was washed with ethanol three times, collected by centrifugation and dried in a vacuum oven at 60 °C for 12 h.

Preparation of CdS/Co-BDC: It was prepared according to the literature method.^15^ First, a precursor solution was obtained by dispersing 1.5 mmol of thioacetamide (TAA) in 0.5 mL of aqueous Cd (NO_3_)_2_ solution and placed in an ice-water bath (10 °C). Then, 100 mg of Co-BDC was completely dispersed in 20 mL of *n*-hexane and sonicated for 20 min, and was subsequently placed in the same ice-water bath as the precursor solution. The precursor solution was added to the dispersed hexane at a rate of 10 μL min^−1^ and stirred rapidly for 2 h. Then, the suspension was treated at 40 °C and then held at 90 °C for 12 h. The final product was filtered several times, washed with ethanol and deionized water, and then vacuum dried at 150 °C for 12 h. By varying the addition of raw materials during the preparation of CdS, pure CdS and CdS/Co-BDC with different CdS loadings were prepared, composite samples (12%, 18%, 23% and 28%). The bare CdS without adding Co-BDC was prepared following the same procedure.

### 2.3. Characterization of photocatalysts

Morphological and structural characterization: JSM-7100F field emission scanning electron microscope (SEM) was used to observe the surface morphology and elemental distribution of the material, and then the microstructure of the material was characterized on a JEOL JEM 2100F high-resolution transmission electron microscope (TEM). X-ray diffraction (XRD) was measured using a Shanghai Shimadzu company's XRD-7000 X-ray diffractometer to determine the XRD pattern of the material, and the crystalline structure was analyzed according to standard cards. The XRD pattern of the materials was used to analyze the crystalline phase structure of the materials based on standard cards. The test conditions were set as follows: X-ray target source was Cu-Kα, the operating voltage was 40.0 kV, the scanning range was from 5° to 80°, and the scanning speed was 2° min^−1^. X-ray photoelectron spectroscopy (XPS) was used to characterize the XPS spectra of the materials using a Thermo Scientific ESCALAB 250 instrument and to analyze the elemental composition of the material surface. UV-Vis diffuse reluctance spectra (DRS) were tested using a TU-1810 spectrophotometer, and the measured UV-visible diffuse reflectance spectra were converted to absorption spectra by the Kubelka–Munk formula.

### 2.4. Photocatalytic reduction of CO_2_

The CO_2_ photocatalytic reduction experiments were carried out in an all-glass automatic on-line trace gas analysis photoreactor (Labsolar-6A, Beijing Perfect Light). The photoreactor was connected to a gas circulation system (Perfect Light LabSolar-6A plus). First, 10 mg of the photocatalyst was evenly distributed on a Petri dish, and the dish and water were placed into the reactor. Then, the reactor was evacuated and purged with high-purity CO_2_ gas three times to remove the air. The reactor was then filled with high-purity CO_2_ gas. The reactor was irradiated with a 300 W xenon lamp (with a cut-off filter, *λ* ≥ 420 nm), and visible light was obtained through a 420 nm cut-off filter. The system automatically collected the gas from the reactor after a period of reaction and analyzed it by an on-line gas chromatograph (GC 9790 II) equipped with a flame ionization detector (FID). Quantification was performed using an external standard method.

### 2.5. Electrochemical testing

The electrochemical impedance (EIS) spectra, transient current response (*i*–*t*) curves and Mott–Schottky curves of the materials were determined using a CHI-760E electrochemical workstation from Shanghai Chenhua Company. The electrochemical workstation consists of a standard three-electrode system with a conductive glass coated with a catalyst sample as the working electrode, Ag/AgCl as the reference electrode, a platinum sheet electrode as the counter electrode, and 0.1 M Na_2_SO_4_ solution as the electrolyte.

The working electrode was prepared as follows: 5 mg of catalyst was added to a mixed solution containing 30 μL of Nafion (5 wt%) and 200 μL of ethanol, and a homogeneous solution was obtained after ultrasonic dispersion for 30 min. Then, 5 μL of the solution was pipetted and dropped on a washed and dried conductive glass with an effective area of 1.0 cm^2^ and finally dried under an infrared lamp several times.

### 2.6. Electron paramagnetic resonance testing

Electron paramagnetic resonance (EPR) test: 5,5-dimethyl-1-pyrroline-*N*-oxide (DMPO) was used as the free radical trapping agent, and the free radicals generated during the photocatalytic process were further detected using the EPR 200M Electron Paramagnetic Resonance Spectrometer of GSI Quantum. Water and methanol were used as solvents to detect ˙OH radicals and ˙O_2_^−^ radicals, respectively.

## 3. Results and discussion

### 3.1. Characterization of samples

**Fig. 1:**
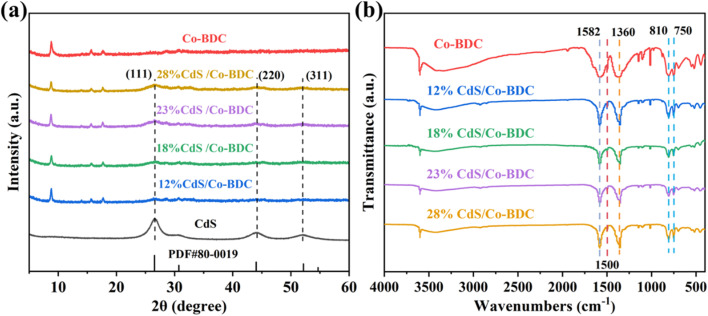
(a) XRD patterns of CdS, Co-BDC and CdS/Co-BDC composites with different weight ratios; (b) FT-IR spectra of the Co-BDC and CdS/Co-BDC composite.

X-ray diffraction (XRD) was used to characterize the crystal structure of the sample. As shown in Fig. 1a, the XRD pattern of Co-BDC displayed several diffraction peaks at 8.8°, 14.1°, 15.7° and 17.8°, corresponding to the (200), (001), (201), and (400) crystal planes of the laminar structure, respectively.^16,17^ Apart from the characteristic reflections of pure Co-BDC, the CdS/Co-BDC showed additional peaks at 26.5°, 44.0° and 52.1°, which corresponded to (111), (220) and (311) crystal planes of CdS, respectively (JCPDS No. 80-0019).^18–20^ Moreover, the intensity of the diffraction peaks ascribed to CdS also increased gradually with increasing content of CdS. It indicated that a heterojunction was formed between CdS and Co-BDC.^21,22^

Fourier transform infrared (FT-IR) spectra were also applied to characterize the structures of CdS/Co-BDC. As displayed in Fig. 1b, the composites with different content of CdS presented nearly identical characteristic absorption peaks with Co-BDC. Generally, the tensile vibration absorption peaks at 750 cm^−1^ and 810 cm^−1^ can be assigned to C–H bond flexural vibrations on the benzene ring of the organic ligand.^23^ The absorption peaks at 1360 cm^−1^, 1500 cm^−1^, and 1582 cm^−1^ were derived from symmetrical vibrations of –COO–, C=O and C=C telescopic vibrations, respectively.^17,24^ The results indicated that the Co-BDC retained its structural properties after being composed with CdS.

**Fig. 2:**
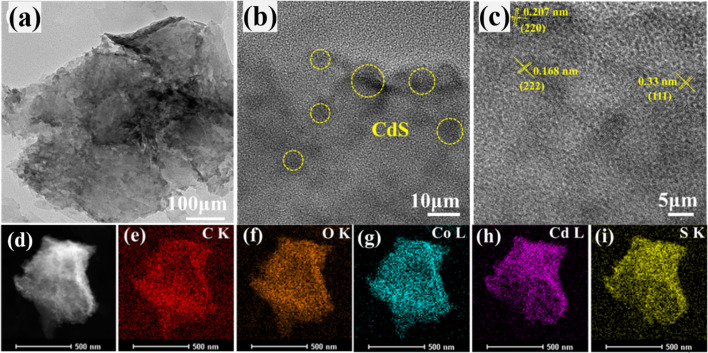
TEM and HRTEM images (a–d) and elemental mappings (e–i) of the 18% CdS/Co-BDC composite.

The surface morphology and microstructure of all the as-synthesized photocatalysts were characterized using a scanning electron microscope (SEM). As shown in Fig. S1a.† CdS exhibited typical spherical particle morphology, while Co-BDC was in the form of a block with some nanosheets (Fig. S1b†). In the SEM images of CdS/Co-BDC (Fig. S1c–f†), some small nanoparticles were clearly observed compared to that of the bare Co-BDC, which indicated that CdS nanoparticles were tightly deposited on the surface of Co-BDC. Numerous CdS nanoparticles agglomerated with the content of CdS increasing to 28% in CdS/Co-BDC. TEM and HR-TEM were taken to further investigate the morphology. Fig. 2a and b exhibited that CdS nanoparticles were tightly recombined with Co-BDC and the size of the CdS nanoparticle was about 10 nm. In Fig. 2c, the crystal plane spacings of CdS were measured to be 0.33 nm, 0.21 nm and 0.17 nm, corresponding to (111), (220) and (311) crystal planes, respectively.^25^ In Fig. 2d–i, the energy dispersive X-ray spectroscopy (EDS) element mapping images revealed that Co, Cd, and S elements were uniformly distributed in 18% CdS/Co-BDC composites, the results were consistent with the results by EDX mapping from SEM shown in Fig. S1g–j.†

### 3.2. Optical characterization

**Fig. 3:**
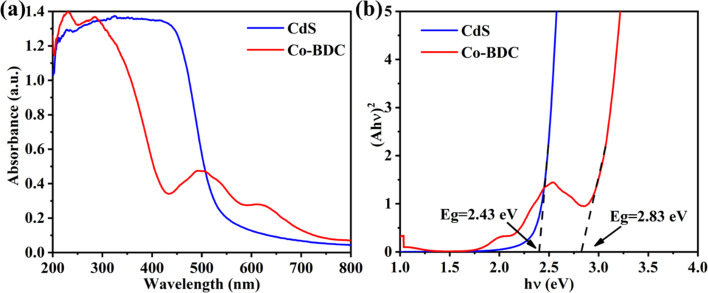
UV-vis of CdS and Co-BDC (a); band curve map of CdS and Co-BDC (b).

The Kubelka–Munk (KM) method was adopted to analyze the optical absorption properties for photoreduction CO_2_.^26^ As shown in Fig. 3a, the absorption edge of the bare CdS was 550 nm. The pure Co-BDC material exhibited absorption in both the UV and visible regions with an absorption band at 500 nm. The band gap energy can be calculated from the Kubelka–Munk function:^27^*αhν* = *A*(*hν* − *E*_g_)^*n*/2^. Where *α* is the absorption coefficient, *h* is Planck's constant, *ν* and *A* represent the optical frequency and proportionality constant. *n* is taken as 4 on account of the indirect bandgap semiconductor of CdS and Co-BDC.^28^Fig. 3b shows the corresponding Tauc plots and the bandgaps (*E*_g_) calculated by the plot of (*αhν*)^2^*versus* the photoenergy (*hν*).^29^ The estimated *E*_g_ values of CdS and Co-BDC are 2.43 eV and 2.83 eV, respectively.

**Fig. 4:**
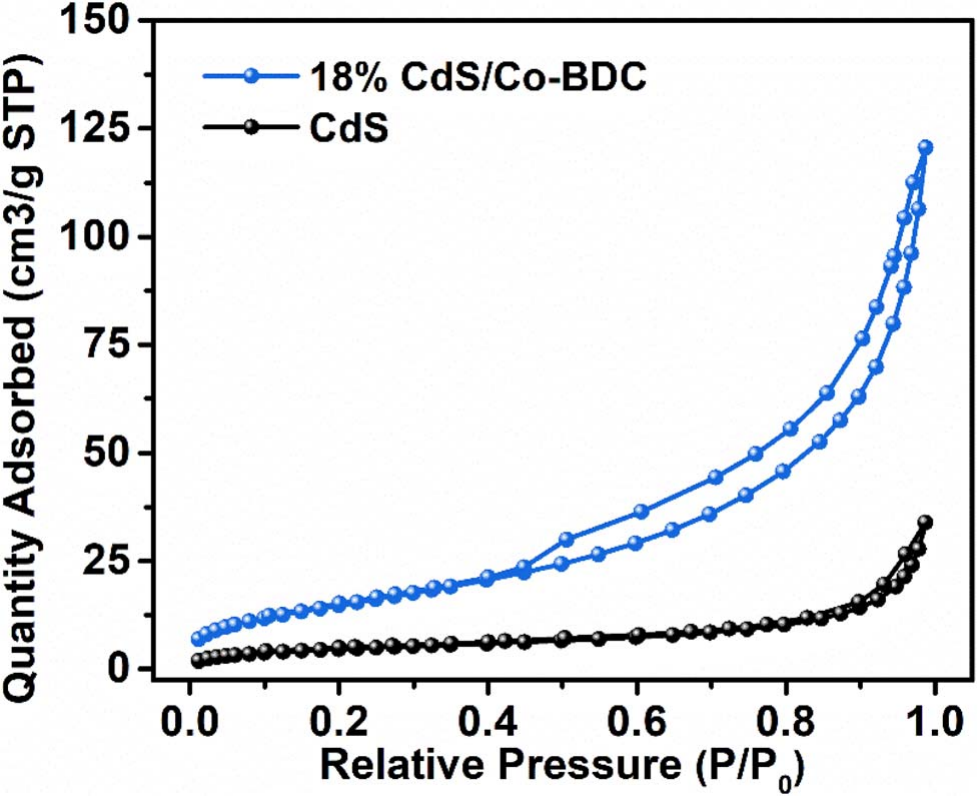
N_2_ adsorption–desorption isotherms of CdS and the 18% CdS/Co-BDC composite.

In the solid–gas system reaction system, the specific surface area is crucial to initiate the photocatalytic reaction. Therefore, N_2_ adsorption/desorption tests were carried out. As shown in Fig. 4 and S2,† the 18% CdS/Co-BDC composites show typical type-IV isotherms, indicating the existence of mesopores (2–50 nm).^30^ When the relative pressure was (0.8 < *P*/*P*_0_ < 1.0), type-H_3_ hysteresis loops appeared, indicating that there may be sheet granular materials. In comparison with the BET surface area and the pore volume of CdS/Co-BDC and CdS (Table S1†), there was a considerable rise of the surface area from 17.39 cm^2^ g^−1^ to 57.42 cm^2^ g^−1^, and the pore volume increased from 0.05 cm^3^ g^−1^ to 0.19 cm^3^ g^−1^. The specific surface area of the prepared CdS/Co-BDC gradually increased with the addition of CdS, which could be attributed to Co-BDC. Moreover, CO_2_ adsorption over CdS and 18% CdS/Co-BDC was also investigated and the results are shown in Fig. S3 and Table S2.† Interestingly, 18% CdS/Co-BDC (4.43 cm^3^ g^−1^) exhibited a higher adsorption capability toward CO_2_ compared to CdS (3.28 cm^3^ g^−1^). The enrichment of the adsorption capability toward CO_2_ boosted its photocatalytic performance for CO_2_ reduction, on account that CO_2_ adsorption was a vital step for CO_2_ reduction. As a consequence, CdS not only increased the specific surface area but also boosted the CO_2_ adsorption of 18% CdS/Co-BDC.

### 3.3. Photocatalytic reduction of CO_2_

**Fig. 5:**
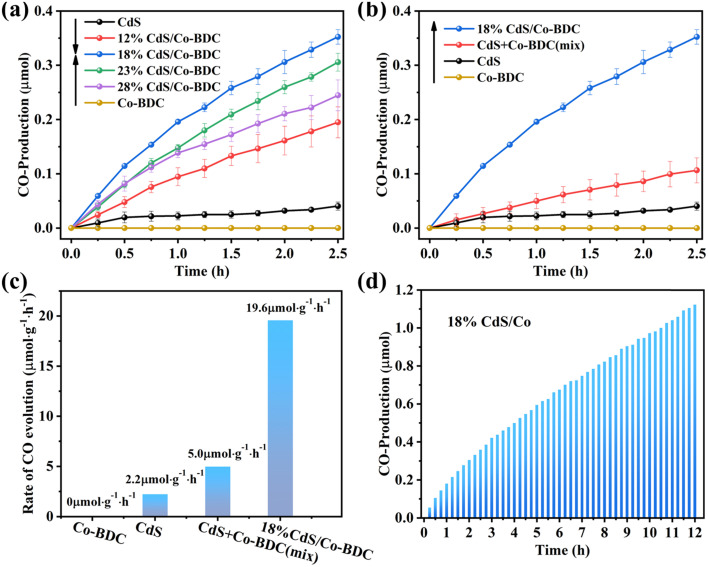
(a) CO production over CdS, Co-BDC and CdS/Co-BDC composites with different weight ratios with CdS; (b) CO production over CdS, Co-BDC, the mixture of CdS and Co-BDC with a ratio of 18% and 18% CdS/Co-BDC composite; (c) CO-evolution rates over CdS, Co-BDC, the mixture of CdS and Co-BDC with a ratio of 18% and 18% CdS/Co-BDC composites; (d) CO production for 12 h on the 18% CdS/Co-BDC composite. All reactions were performed under visible light.

The photocatalytic performance of the as-synthesized samples was evaluated by photoreduction of CO_2_ under visible irradiation (*λ* ≥ 400 nm). As shown in Fig. 5a, Co-BDC exhibited no photocatalytic CO_2_ reduction activity. The optimized photocatalytic CO_2_ reduction performance was achieved over 18% CdS/Co-BDC with a CO evolution rate of 19.6 μmol g^−1^, which was about 8.9 times than that of pure CdS.

Also, the physical mixture of CdS and Co-BDC with a weight ratio of 18% was also tested for photoreduction of CO_2_ under the same condition (Fig. 5b). It also showed that the CO yield of the physical mixture catalysts was also higher than those of the pure CdS and Co-BDC catalysts, while they were lower than that of 18% CdS/Co-BDC. It could be ascribed to the synergistic effect between CdS and Co-BDC. The physical mixture of CdS and Co-BDC shows a lower synergistic effect, owing to lower contact between the CdS and Co-BDC. The CdS/Co-BDC composites were prepared to insert CdS into the cavities of Co-BDC by the impregnation method, the composites obtained a much bigger contact of CdS and Co-BDC, which showed a higher synergistic effect between CdS and Co-BDC. Also, the stability of 18% CdS/Co-BDC was also tested, as shown in Fig. 5d; it exhibited a stable conversion of CO_2_ under visible irradiation (*λ* ≥ 400 nm) for 600 min. To further confirm the stability of the photocatalyst, the structure of the used photocatalyst was analyzed by XRD and FTIR. Fig. S5† shows XRD patterns and FT-IR spectra of the fresh and used photocatalyst, the peaks of the used composite CdS/Co-BDC were consistent with those of the fresh sample, which showed that the CdS/Co-BDC composites are stable.

### 3.4. Photocatalytic mechanism

**Fig. 6:**
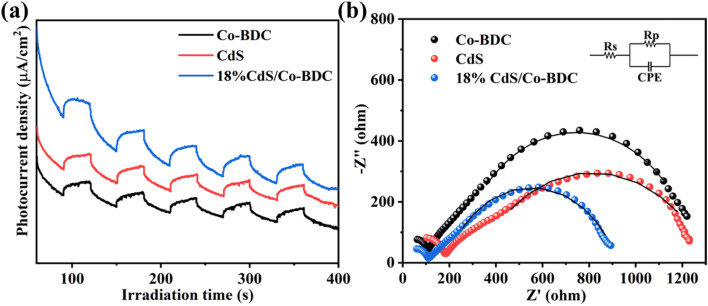
Transient photocurrent responses (a) and EIS Nyquist plots (b) of the Co-BDC, CdS and 18% CdS/Co-BDC composite.

The separation and interfacial transfer of charge carriers have an effect on photocatalytic performance. Therefore, transient photocurrent responses of pure Co-BDC, CdS and 18% CdS/Co-BDC were recorded in several on–off cycles of light irradiation. As displayed in Fig. 6a, the photocurrent density reached a constant value with light irradiation turned on, while the photocurrent density dropped to a lower value with the light turned off. Obviously, the photocurrent density of 18% CdS/Co-BDC was higher than that of CdS and Co-BDC. The result can be attributed to the effective separation of photo-induced electron–hole pairs generated by the heterostructure of CdS and Co-BDC. In addition, electrochemical impedance spectroscopy (EIS) was conducted to investigate the resistance behavior of the charge transfer for the various samples, as shown in Fig. 6b and Table S3.† It is evident that 18% CdS/Co-BDC displayed a smaller resistance (735.5 Ω) than CdS (1006 Ω) and Co-BDC (1012 Ω). It suggested that the 18% CdS/Co-BDC obtained high charge transfer efficiency.^31^ To further confirm the charge transfer property, the time-resolved PL (TR-PL) spectra were used to observe photo-generated charge carriers with an emission peak at 457 nm. The average emission lifetime (*τ*) can be determined using the following equation:^32^*τ* = (*B*_1_*τ*_1_^2^ + *B*_2_*τ*_2_^2^ + *B*_3_*τ*_3_^2^)/(*B*_1_*τ*_1_ + *B*_2_*τ*_2_ + *B*_3_*τ*_3_)where *B*_1_, *B*_2_ and *B*_3_ mean the weight factors, and *τ* represents the fluorescence lifetime. As shown in Fig. S6 and Table S3,† the average lifetime of 18% CdS/Co-BDC (4.0 ns) is longer than that of Co-BDC CN NSs (3.1 ns) and CdS (3.8 ns). It means the obtained composites enhanced the efficiency of the photo-generated electron–hole separation.

**Fig. 7:**
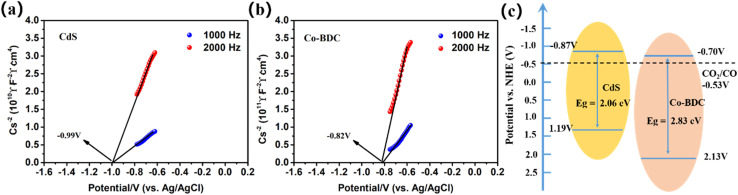
Mott–Schottky plot: (a) CdS, (b) Co-BDC, and (c) the proposed schematic illustration of the energy-level configuration.

Furthermore, the electrochemical Mott–Schottky (M–S) plots of the as-prepared samples were measured to investigate the bandgap structures. As shown in Fig. 7, the samples displayed positive slopes, indicating that all the samples are n-type semiconductors. The flat-band potentials of CdS and Co-BDC were calculated to be −0.99 V *vs.* Ag/AgCl electrode (*i.e.*, −0.77 V *vs.* NHE, pH = 7) and −0.82 V *vs.* AgCl electrode (*i.e.*, −0.60 V *vs.* NHE, pH = 7). After the calibration and calculation, the conduction band (CB) and value band (VB) of CdS were estimated to be −0.87 V and 1.19 V, respectively. The LUMO and HOMO of Co-BDC were calculated to be −0.70 V and 2.13 V, respectively. According to the energy-level structure in Fig. 7c, it was obvious that the CB position of CdS was more negative than the LUMO of Co-BDC, and the VB of CdS was less positive than the HOMO of Co-BDC.

**Fig. 8:**
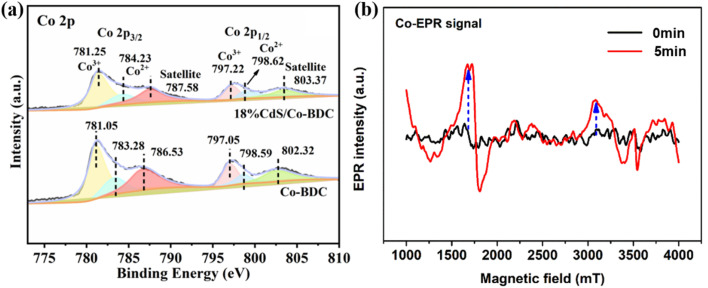
(a) XPS spectra of CdS, Co-BDC and 18% CdS/Co-BDC composites and (b) EPR spectrum of Co(ii) in CdS/Co-BDC measured at 298 K.

The chemical composition and chemical state of elements in 18% CdS/Co-BDC was characterized by X-ray photoelectron spectroscopy (XPS). As shown in Fig S4,† the survey spectrum presented elemental peaks of C, O, Cd, S, and Co, which is consistent with the mapping results. The main peaks at 798 and 782 eV in the Co 2p spectrum of Co-BDC could be assigned to Co 2p_1/2_ and Co 2p_3/2,_ respectively^33^ (Fig. 8a). Two peaks located at 783.28 eV and 798.59 eV corresponded to Co^2+^ in Co-BDC,^34^ another group of peaks located at 781.05 eV and 797.05 eV were identified as Co^3+^ species in Co-BDC.^35,36^ Meanwhile, compared with that of Co-BDC, the Co 2p peaks of the 18% CdS/Co-BDC composite exhibited positive shift (−1 eV), which means the charge of Co-BDC prefers to obtain the charge from CdS in the CdS/Co-BDC composite, which revealed the existence of charge interactions between CdS and Co-BDC in the composites. Electron paramagnetic resonance (EPR) was used to study the metal valence changes through the unpaired electrons in the samples. Co(ii) with a single-electron pair could detect the EPR signal, as its electron arrangement formula is [Ar]3d^7^4s^2^. As shown in Fig. 8b, only a weak signal can be detected by EPR, owing to the low content of Co(ii) in the 18% CdS/Co-BDC.^37^ It is noteworthy that the Co(ii) signal was enhanced after 5 min of illumination and it is attributed to the large amount of Co(ii) species, which are derived from Co(iii). These results indicated that the photo-excitation electrons could transfer from CdS to Co(iii).

**Fig. 9:**
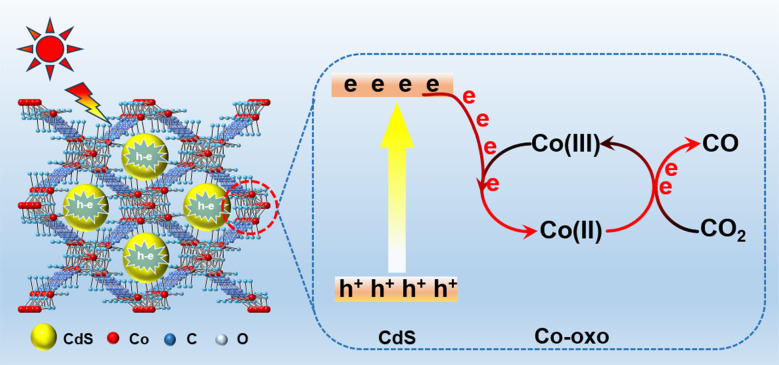
Schematic of CO_2_ conversion over CdS/Co-BDC *via* Co(ii)/Co(iii) redox cycling.

Accordingly, a possible reaction mechanism for photocatalytic CO_2_ reduction over 18% CdS/Co-BDC photocatalyst was proposed, as shown in Fig. 9. The deposition of CdS nanoparticles on the surface of Co-BDC triggered abundant intimate contact between CdS and Co-BDC, and it played an important role in the photocatalytic CO_2_ reduction. Upon the visible light irradiation, electrons and holes are generated in the conduction band (CB) and valence band (VB) over CdS, respectively. The photo-generated electrons of CdS will migrate to the Co-oxo of Co-BDC by the reduction of Co(iii)-oxo to Co(ii)-oxo,^38^ and the electrons are transferred to CO_2_ adsorbed on Co-oxo active sites to produce CO by the transition of Co(ii)-oxo to Co(iii)-oxo. As a result, an enhanced photocatalytic CO_2_ reduction performance is achieved over 18% CdS/Co-BDC by Co(ii)/Co(iii) redox cycling.

## 4. Conclusions

Overall, Co-BDC materials were synthesized using room temperature stirring, and a series of CdS/Co-BDC composites with different ratios were synthesized by the two-solvent method, which was used for the study of photocatalytic CO_2_ reduction. With the increase of CdS content, the morphology of the composites gradually changed from the initial flake-like morphology to surface-loaded CdS spherical nanoparticles. CdS/Co-BDC synthesized by using the dual-solvent method resulted in a more contact area between CdS and Co-BDC, leading to stronger interactions on the surface than CdS. In addition, the photocatalytic CO_2_ reduction performance of the composites was greatly improved under visible light irradiation, with the 18% CdS/Co-BDC composite showing the best photocatalytic CO_2_ reduction performance, which was higher than that of the pure CdS and the mixtures of CdS and Co-BDC, with a CO production rate of 19.6 μmol g^−^1 h^−1^, which was higher than that of the pure CdS (2.2 μmol g^−1^·h^−1^) by about 9 times. The photocatalytic mechanism study showed that when CdS was complexed with Co-BDC, electrons were transferred from CdS to Co-BDC by Co(ii)/Co(iii) redox cycling, which improved the carrier separation efficiency and enhanced the photocatalytic reduction activity of CO_2_. This work may provide a novel strategy to enhance the photocatalytic activity by metal oxide clusters of other MOFs and it also may provide some guidance for further improving the separation efficiency of charge carriers by two kinds of metal oxide clusters of MOFs.

## Data availability

The authors confirm that the data supporting the findings of this study are available within the article and its ESI.†

## Author contributions

Ruina Xuan, and Jieqiong Mo: investigation, data curation, analysis of data, writing – original draft preparation. Jiwen Chen: soft of material characterization. Yixin Dou: visualization. Zhuo jiang: methodology. Xiaofang Li: language modification. Xiaobo Wang: material characterization. Bo Chai: supervision and material characterization. Chunlei Wang: methodology and writing review. Deng Ding: conceptualization, methodology, financial support, writing – reviewing and editing, resources. Juntao Yan: financial support, writing – reviewing and editing.

## Conflicts of interest

There are no conflicts to declare.

## Supplementary Material

RA-014-D4RA04842C-s001
