# Supplementary material for: Enhanced photocatalytic CO2 conversion of a CdS/Co-BDC nanocomposite via Co(ii)/Co(iii) redox cycling
Source: RSC Adv. 2024 Aug 13;14(35):25247–55. doi: 10.1039/d4ra04842c (PMC11320058; doi:10.1039/d4ra04842c)
Supplement: RA-014-D4RA04842C-s001 [file RA-014-D4RA04842C-s001.pdf]

Supplementary Information (SI) for RSC Advances.
This journal is © The Royal Society of Chemistry 2024

Supporting Information

Enhanced photocatalytic CO_2 conversion of CdS/Co-BDC nanocomposite via Co (II)/Co (III) redox cycling

[a,c]*Ruina Xuan,* [a,c]*Jieqiong Mo,* [a]*Jiwen Chen,* [a]*Yixin Dou,* [a]*Xiaofang Li,* [b]*Zhuo jiang,* [a]*Bo Chai,* [a]*Chunlei Wang,* [a]*Deng Ding*,* [a]*Juntao Yan*,* [a]*Xiaobo Wang**

a College of Chemistry and Environmental Engineering, Wuhan Polytechnic University, Wuhan 430023, China

b School of Electrical Engineering and Automation, Wuhan University, Wuhan 430072, China

Corresponding Author: Deng Ding

c These authors contributed equally: *Ruina Xuan and Jieqiong Mo*.

Address: College of Chemistry and Environmental Engineering, Wuhan Polytechnic University, Wuhan 430023, China

E-mail: dingdeng@whu.edu.cn

Phone: 0086-15927216471

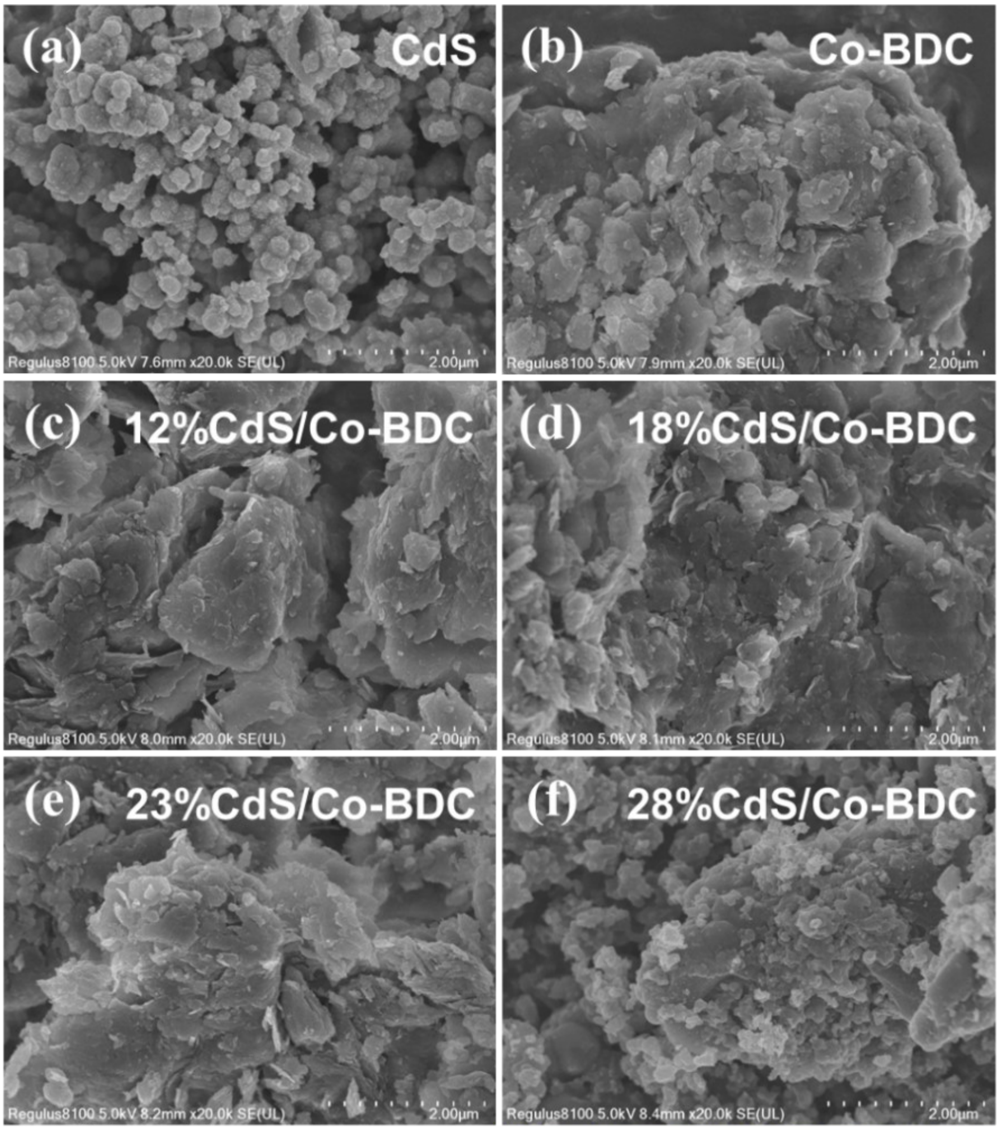
(a) CdS
(b) Co-BDC
(c) 12%CdS/Co-BDC
(d) 18%CdS/Co-BDC
(e) 23%CdS/Co-BDC
(f) 28%CdS/Co-BDC
Regulus8100 5.0kV 7.6mm x20.0k SE(UL) 2.00μm
Regulus8100 5.0kV 7.9mm x20.0k SE(UL) 2.00μm
Regulus8100 5.0kV 8.0mm x20.0k SE(UL) 2.00μm
Regulus8100 5.0kV 8.1mm x20.0k SE(UL) 2.00μm
Regulus8100 5.0kV 8.2mm x20.0k SE(UL) 2.00μm
Regulus8100 5.0kV 8.4mm x20.0k SE(UL) 2.00μm

Fig. S1. SEM images of CdS (a); Co-BDC (b); 12% CdS/Co-BDC (c); 18% CdS/Co-BDC (d); 23% CdS/Co-BDC (e); 28% CdS/Co-BDC(f); 18% CdS/Co-BDC composite (g-j).

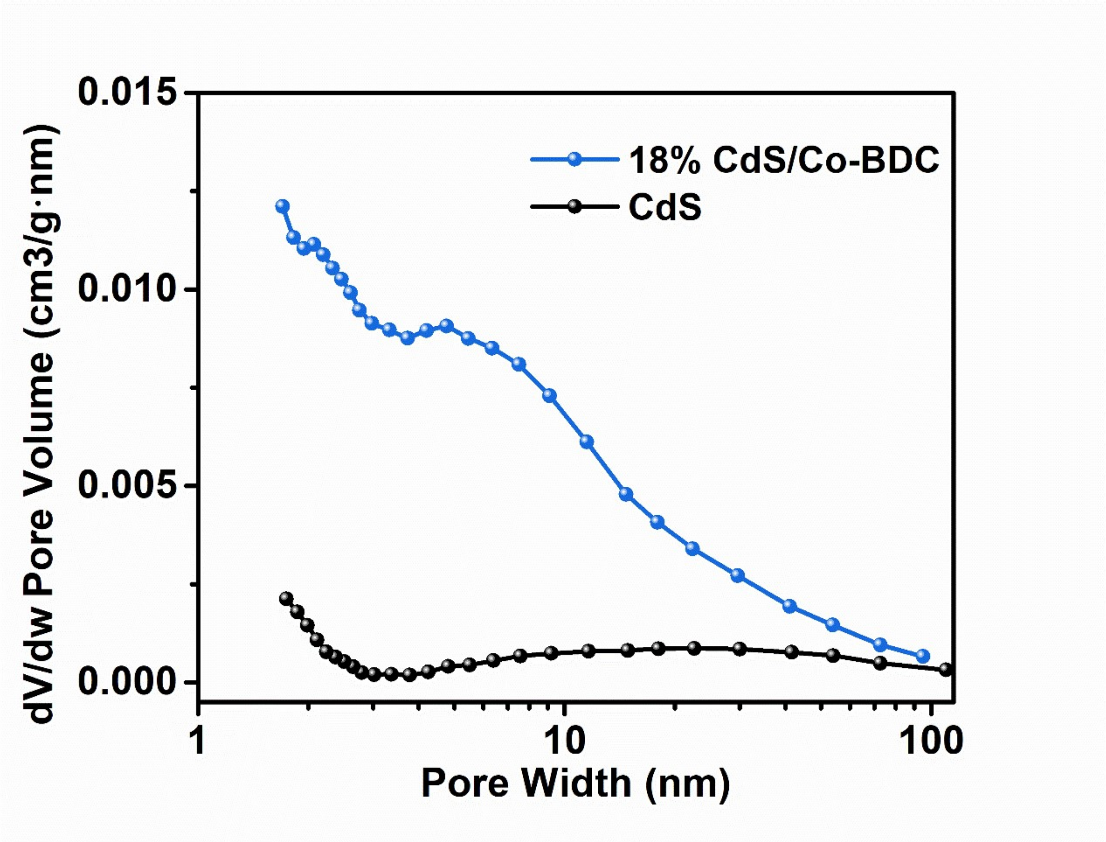

Fig. S2. N_2 The pore size distributions of CdS, Co-BDC and 18% CdS/Co-BDC composite.

Table S1 Specific surface area and pore structure parameters of materials

Sample	S_{BET} ($m^2 \cdot g^{-1}$)	Pore size (nm)	Pore volume ($cm^3 \cdot g^{-1}$)
CdS	17.39	14.71	0.05
18%CdS/Co-BDC	57.42	9.94	0.19

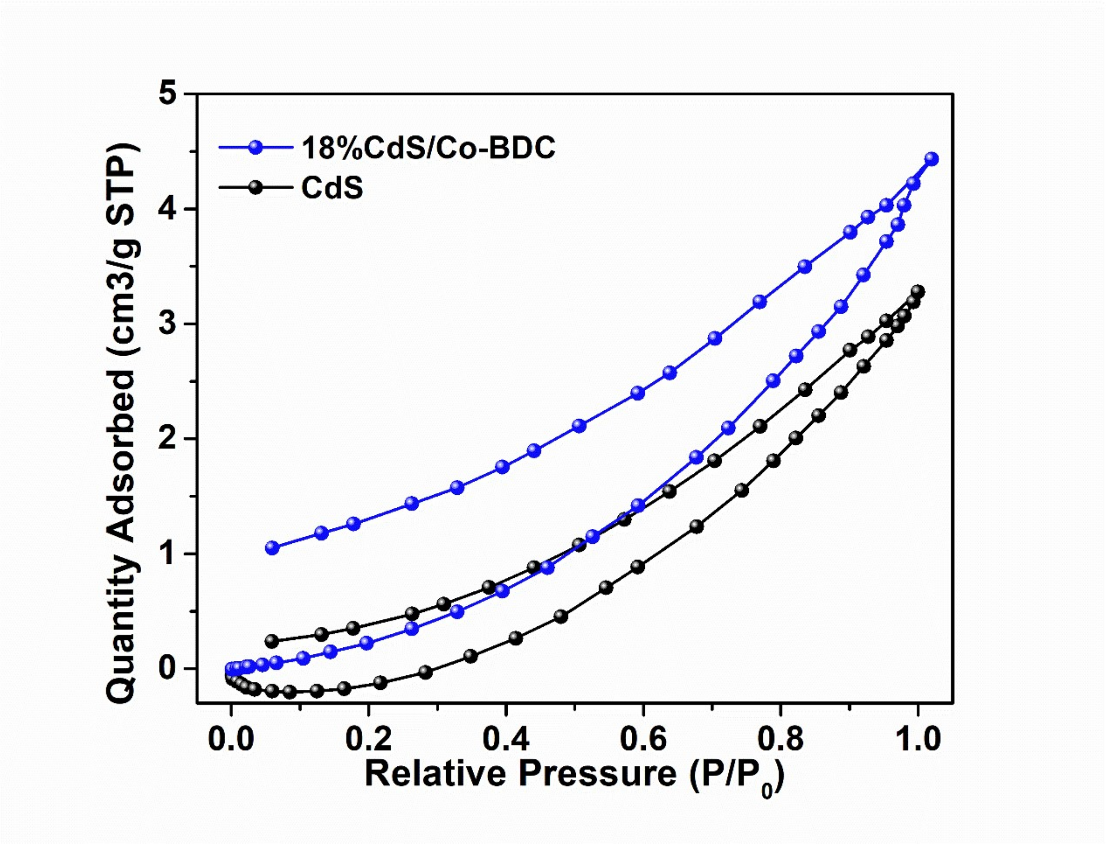

Fig. S3. CO_2 adsorption curve of CdS, Co-BDC and 18% CdS/Co-BDC composite at 298 K.

Table S2 CO_2 adsorption-desorption isotherms of CdS, Co-BDC and 18% CdS/Co-BDC composite

Sample	CO-production (μmmol·g^{-1}·h^{-1})	CO_2-quantity absorbed (cm^3·g^{-1})	CO-production/CO_2-quantity absorbed (μmmol·cm^{-3}·h^{-1})
CdS	2.2	3.28	0.67
18%CdS/Co-BDC	19.6	4.43	4.42

Table S3 The fitted Rct values of Co-BDC, CdS and 18% CdS/Co-BDC composite

Sample	Rp（Ω）
CdS	1006
Co-BDC	1012
18% CdS/Co-BDC	735.5

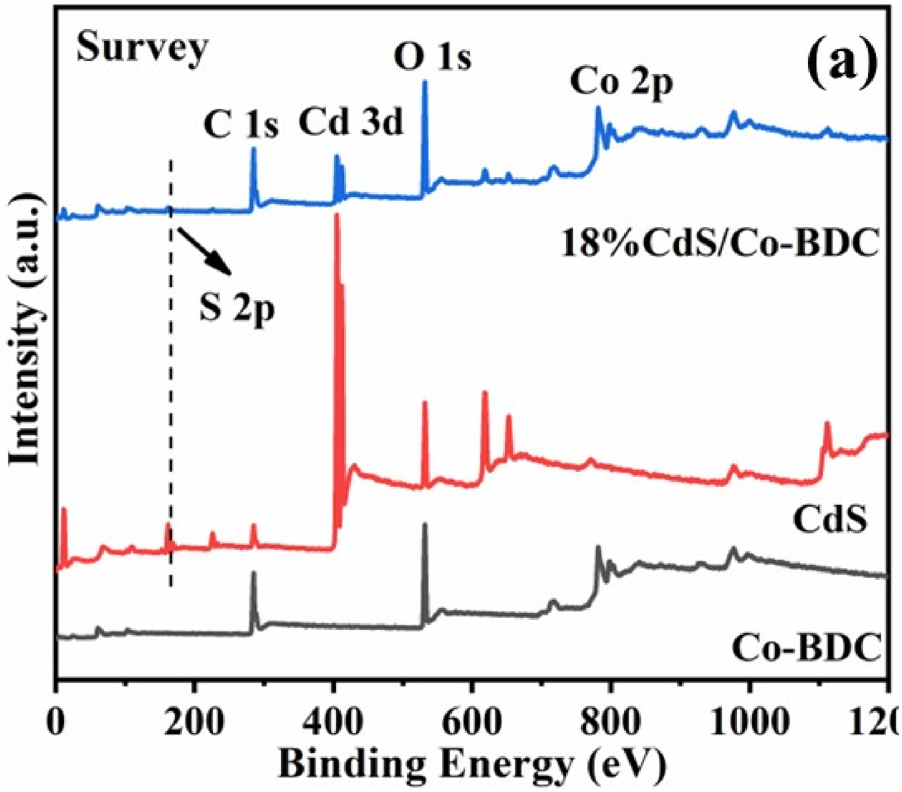

Fig. S4. XPS spectra of survey scan of CdS, Co-BDC and 18% CdS/Co-BDC.

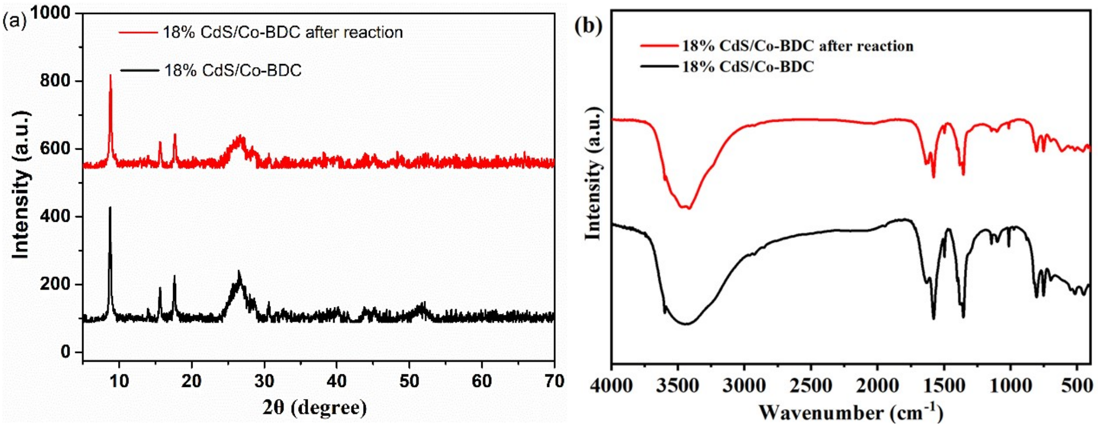

Fig. S5 XRD and FT-IR pattern of fresh and used 18%CdS/Co-BDC nanocomposites.

Fig. S5 **XRD and FT-IR pattern of fresh and used 25-Bi_2O_2Se/BiOCl nanocomposites.**

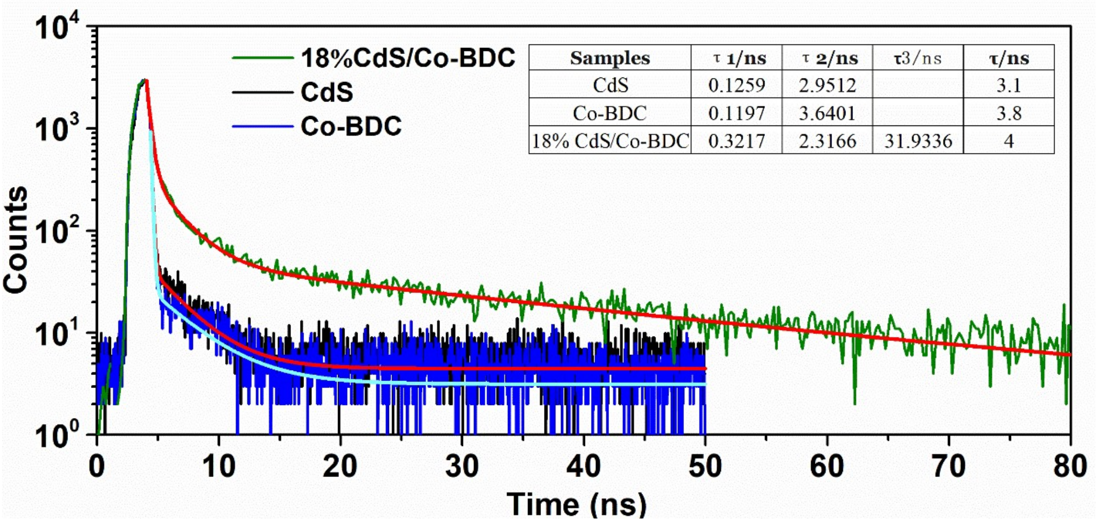

Fig. S6. Time-resolved PL spectra of bare CdS, Co-BDC and 18% CdS/Co-BDC.

Table S4. Analyses of the time-resolved transient PL decay spectra of the samples.

Samples	τ1/ns	B1	τ2/ns	B2	τ3/ns	B3	τ/ns
CdS	0.1259	1278.42	2.9512	38.49			3.13
Co-BDC	0.1197	1349.10	3.6401	23.40			3.86
18% CdS/Co-BDC	0.3217	4703.53	2.3166	354.08	31.9336	0.98	4.0
